# An explainable machine learning model for predicting osteoporotic fragility fractures: a retrospective study in South China

**DOI:** 10.3389/fmed.2026.1869681

**Published:** 2026-06-19

**Authors:** Zebing Si, Konghe Hu, Huajun Wang, Xiaofei Zheng

**Affiliations:** 1 Department of Sports Medicine, Jinan University, Guangzhou, Guangdong, China; 2 Department of Orthopedics, Yuebei People's Hospital, Shaoguan, Guangdong, China

**Keywords:** feature selection, fragility fracture, machine learning, model, osteoporosis

## Abstract

**Background:**

Osteoporotic fragility fractures often lead to hospitalization and impose a significant economic burden. To investigate the clinical characteristics of hospitalized major osteoporotic fractures in South China.

**Methods:**

In this study, we identified 1125 hospitalized patients with osteoporotic fractures who underwent DXA examination at the Yuebei People's Hospital between January 1, 2019, and December 31, 2025. From these patients, we collected 73 features, including clinical, laboratory, and radiological features. Recursive Feature Elimination (RFE) was applied to filter these features. Then we evaluated six machine learning algorithms (XGBoost, Random Forest, Support Vector Machine, CatBoost, LightGBM, and Logistic Regression) to construct the best predictive model. In order to explain the model, SHAP (SHapley Additive exPlanations) was used to assess the importance of each attribute.

**Results:**

LightGBM achieved the highest training-set ROC-AUC (0.903) and the highest independent test-set ROC-AUC (0.840) among the six models. Through SHAP analysis, we observed that age, Hepatitis C virus IgG antibody, and serum sodium were identified as the top factors contributing to increased fracture risk.

**Conclusion:**

Our model has a good ability of predicting osteoporotic fragility fractures. This paves the way for earlier intervention in patients who are susceptible to osteoporotic fragility fractures.

## 1 Introduction

Osteoporosis is characterized by a reduction in bone density, which often leads to an increased risk of fractures (1). Fractures not only induce severe pain in patients but also impose physical limitations, thereby threatening quality of life (2). Recently, as the incidence of osteoporotic fragility fractures grows, this trend poses a threat to public health and increases the socioeconomic burden. As people get older, bone strength often wanes, raising the odds of fragility fractures (3). Bone mineral density (BMD) acts as the most important factor for assessing skeletal resilience. Still, low BMD doesn't guarantee a break, while other factors like falls, muscle loss, or underlying conditions frequently tip the scales too (4). Treating all Osteoporotic patients with pharmacotherapy indiscriminately is frequently unfeasible. Identifying the specific patient subgroups who are truly most vulnerable to fracture remains a significant challenge (5). Therefore, it is essential to construct a predictive model for osteoporotic fragility fractures that can help patients receive precision therapy (6, 7).

In recent years, many research groups have worked on developing predictive models to identify people at risk of fragility fractures (7). The Fracture Risk Assessment Tool (FRAX) is still the most widely used tool for estimating the 10-year risk of osteoporotic fractures (8). However, FRAX still has notable limitations that can reduce its predictive accuracy. It mainly uses basic demographic data and a small number of yes/no clinical risk factors. These do not fully capture the complex biology behind bone fragility and fracture risk (9). Also, because FRAX was designed for populations rather than individuals, it may not accurately predict risk at the patient level (9–11). To address these limitations, researchers are adding new predictive factors not included in FRAX and testing machine learning (ML) methods alongside traditional statistical approaches.

Machine learning (ML) has attracted growing interest in disease research because it may improve fracture risk prediction (12–15). Traditional statistical methods often struggle with complex data, such as combining clinical notes, imaging, and lab test results. So in practice, ML can give a risk estimate that's more tailored to the individual patient—not just a one-size-fits-all number (16). For example, Zabihiyeganeh et al. used a wider range of clinical, biochemical, and imaging features to predict future fragility fractures and showed better results than FRAX (17). Similarly, one study developed a balanced bagging model to predict osteoporotic vertebral compression fractures in postmenopausal women using clinical, biological, and musculoskeletal parameters (18). However, explainable ML is still rarely used in this area. This is a challenge because if a model remains opaque, doctors cannot see which factors lead to the prediction, making it hard to trust the results (19).

In this study, we adopted an explainable approach and investigated various ML algorithms to predict the incidence of fragility fractures considering clinical data, laboratory data, and radiology data from Yuebei People's Hospital. The primary objective of this study was to identify the best ML algorithm to predict the incidence of fragility fractures in osteoporosis patients. The secondary objectives were to determine the important features that contribute to this prediction and how these features interact with each other.

## 2 Materials and methods

### 2.1 Data sources and preprocessing

This study, a retrospective case analysis utilizing clinical data, sought to develop and validate a machine learning model to predict fracture risk after low-energy trauma in osteoporosis patients. The data were extracted from the hospital information system of Yuebei People's Hospital, covering the period from January 2019 to December 2025. Initially, all inpatients who underwent dual-energy X-ray absorptiometry (DXA) during the observation period were identified. From this initial group, a final cohort was established by sequentially applying predefined inclusion and exclusion criteria for model development and analysis. It is important to note that no active participant recruitment was conducted for this study.

The diagnosis of osteoporosis was based on the gold standard of bone mineral density (BMD) measurements, with a T-score of ≤ −2.5 at the lumbar spine (L1-L4) and/or hip (including the femoral neck or total hip). Low-energy trauma was defined as any injury resulting from minor activities or light external forces, such as a fall from standing height, a minor collision, or twisting motions. High-energy injuries, such as those caused by traffic accidents, falls from significant heights, or impacts during sports, were excluded from this definition. Fragility fractures were defined according to the World Health Organization (WHO) criteria, which classify them as fractures resulting from minimal trauma that would not typically cause a fracture in healthy bone. The study focused on the low-energy trauma, such as falling from standing height or less. These fractures were initially identified through interviews or questionnaires and later confirmed with X-rays and/or medical reports. A total of 73 baseline variables were considered, including age, sex, vitamin D, and calcium intake, and bone mineral density (measured at the total hip, lumbar spine, and femoral neck).

During data preprocessing, the patient registration number was used as the unique identifier. Duplicate records and invalid cases with missing registration numbers were removed. The variables with more than 30% missing values were excluded. In addition, the proportion of missing values was calculated for each sample across the variables, and cases with more than 30% missing data were excluded. For the remaining missing values, continuous variables were imputed using the median, whereas categorical variables were imputed using the mode.

**Figure 1:**
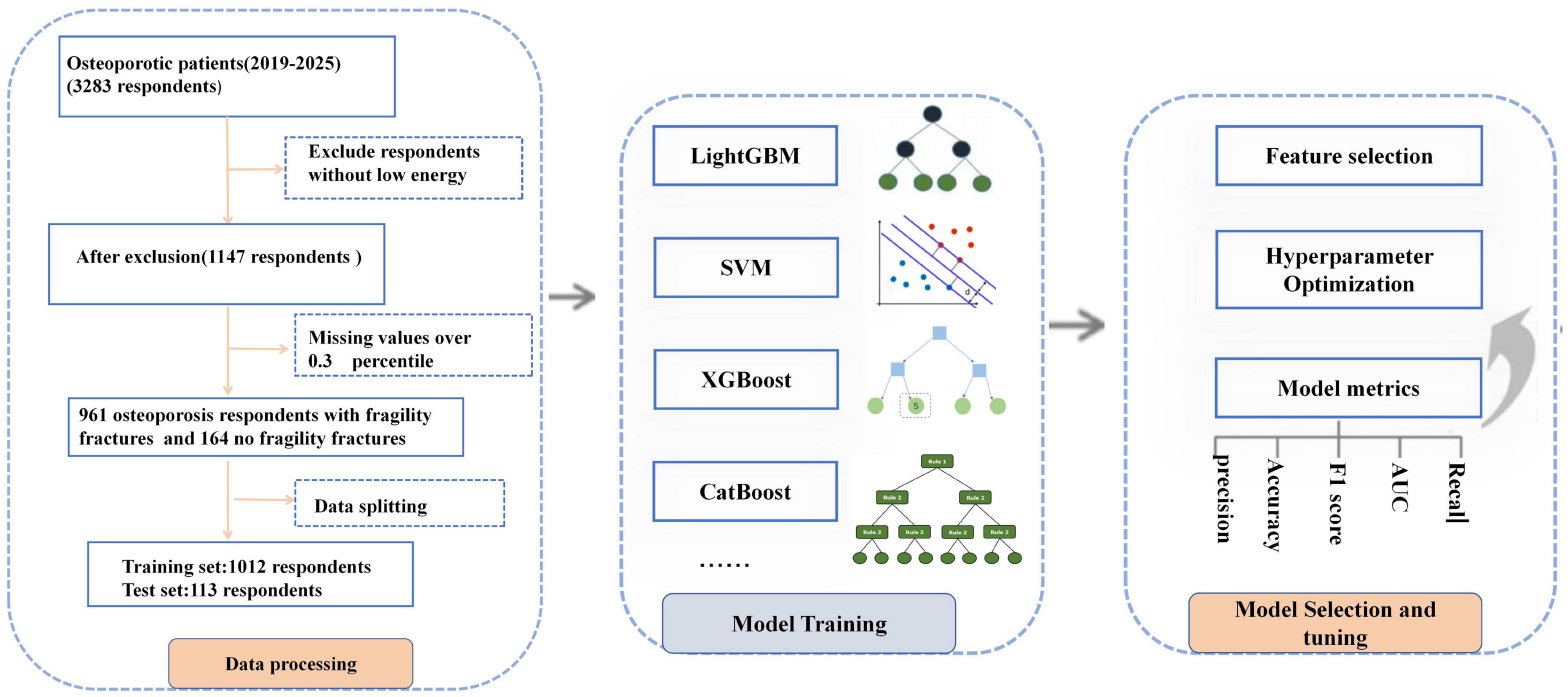
The framework of the study.

The data were split into training and test sets at an 9:1 ratio. In the training set, there were 148 negative cases and 864 positive cases; in the test set, there were 16 negative cases and 97 positive cases. All modeling features had been standardized before model development. Continuous variables were transformed using StandardScaler, with scaling parameters estimated from the training cohort and then applied to the independent test cohort. The test cohort was not used to estimate any scaling parameter, thereby reducing the risk of data leakage. No missing values were detected in the final modeling files, and therefore no additional imputation was required. All subsequent model-development procedures, including feature selection, SMOTE oversampling, hyperparameter tuning, threshold selection, and model fitting, were restricted to the training cohort. The independent test cohort was held out throughout model development and was used only once for final performance evaluation. The pipeline of the model can be seen in Figure 1.

### 2.2 Handling class imbalance with SMOTE

The study employed the SMOTE for data augmentation. Originally designed to address class imbalance by oversampling minority classes, SMOTE can also be applied to augment the minority class. This technique operates within the feature space, generating synthetic minority class instances by interpolating between existing examples. Specifically, it considers each minority instance, identifies its k-nearest neighbors (where k depends on the required oversampling ratio), and synthesizes new data points along the line segments connecting them. The synthesis involves three core operations: first, computing the feature vector difference between the current instance and a selected neighbor; second, scaling this difference by a random value between 0 and 1; and finally, adding the resultant vector to the original instance's feature vector.

To avoid information leakage, SMOTE was applied only within the training portion of each cross-validation fold. The validation folds and the independent test cohort were never oversampled. Thus, synthetic samples were generated exclusively from the training data available within each fold.

### 2.3 Attribute selection

Attribute selection can reduce the risk of overfitting by removing irrelevant or redundant predictors. RFE (Recursive feature elimination) and SelectKBest were compared for feature selection. RFE iteratively removes the least important variables according to the feature-ranking coefficients generated by the base estimator. Whereas SelectKBest, which applies statistical tests, including the ANOVA F-test, to identify the most informative variables. To avoid information leakage, all feature-selection procedures were performed after the initial train-test split and were restricted to the training cohort only. The independent test cohort was not used during feature selection, hyperparameter tuning, or model construction. During cross-validation, RFE and SelectKBest were embedded within the cross-validation pipeline and refitted separately within each training fold; the selected features were then applied to the corresponding validation fold. For final model development, the selected RFE feature panel was derived from the complete training cohort and subsequently applied unchanged to the independent test cohort. Based on the comparative analysis, the RFE-selected feature panel showed better overall performance and was therefore used for subsequent model development. To ensure fair comparison across algorithms, the same RFE-selected feature set was used for all six machine-learning models.

### 2.4 Construction of prognosis predictive model by machine learning

XGBoost grows trees sequentially; in our study this fairly simple mechanism gave a stable model, though the gain depends on how strongly we regularize (20). It optimizes the loss using first- and second-order information, which typically speeds convergence and—at least in our data—improves generalization by locating better minima. We also applied L1/L2 penalties and kept them conservative to reduce overfitting. Random forests aggregate many trees trained on bootstrap samples while restricting candidate features at each split; this lowers inter-tree correlation and slightly improves robustness on noisier records (21). An SVM seeks a maximum-margin hyperplane and, via kernels, attains nonlinear decision functions without explicit feature mapping (22). CatBoost uses ordered target statistics to encode categories and avoid leakage, adding trees with early-stopping on a validation set. LightGBM selects splits by maximal loss reduction (leaf-wise growth), which in our experiments was faster than level-wise methods on larger tables (23). Logistic regression models class probabilities with a sigmoid applied to a linear predictor; we use it when speed and interpretability matter most (24). For completeness, Table 1 lists the hyperparameters we actually used; defaults apply unless stated.

**Table 1:** The parameters of six machine learning algorithms.

ML algorithm	Parameters
XGBoost	n_estimators = 200; learning_rate = 0.02; max_depth = 2; min_child_weight = 10; gamma = 1; subsample = 0.75; colsample_bytree = 0.75; reg_alpha = 2; reg_lambda = 80; tree_method = hist; eval_metric = auc
RandomForest	n_estimators = 700; criterion = gini; max_depth = 4; max_features = sqrt; min_samples_leaf = 70; min_samples_split = 130; bootstrap = True
SVM	'kernel = linear; C = 0.005; gamma = scale; probability = True; tol = 0.001
Catboost	iterations = 180; learning_rate = 0.016; depth = 2; l2_leaf_reg = 90; random_strength = 4; bagging_temperature = 2.5; loss_function = Logloss; eval_metric = AUC
LightGBM	n_estimators = 260; learning_rate = 0.02; num_leaves = 9; max_depth = 3; min_child_samples = 80; min_split_gain = 0.4; subsample = 0.75; colsample_bytree = 0.75; reg_alpha = 4; reg_lambda = 120; objective = binary
Logistic regression	solver = lbfgs; C = 0.005; max_iter = 10000; fit_intercept = True; tol = 0.0001

Hyperparameter tuning was performed for each model. All hyperparameters were optimized exclusively within the training set, while the independent test set was strictly reserved for final model evaluation and was not involved in model selection. For each algorithm, a predefined hyperparameter search space was specified, and candidate parameter combinations were evaluated using stratified cross-validation.

The parameters of six machine learning algorithms were listed in Table 1.

### 2.5 SHAP

To explain the model, we used SHAP to decompose predictions into feature-level contributions and ranked variables by their mean absolute SHAP values (25). For each individual, we report the model score together with its SHAP breakdown, which made case-level discussions more concrete during internal reviews. We mainly show the SHAP summary (beeswarm) plot: features are ordered by mean SHAP, the x-axis is the SHAP value, and point colors indicate the raw feature values, so one can see both ranking and direction at a glance. Overall, SHAP was helpful for seeing the main factors of the model; that said, correlated laboratory variables can split or inflate attributions, so we interpret the rankings with that caveat in mind and inspected several force plots for representative cases as a cross-check (26).

### 2.6 Model evaluation and statistical analysis

Internal generalization performance was estimated using stratified 5-fold cross-validation in the training cohort. Out-of-fold predictions were generated for each model and used to calculate internal AUC, PR-AUC, sensitivity, specificity, balanced accuracy, and confusion matrices. Final models were then trained on the complete training cohort and evaluated once in the independent test cohort.

AUC 95% confidence intervals were estimated using bootstrap resampling with 3,000 iterations. ROC curves, PR curves, confusion matrices, and AUC learning curves were generated for all six models. Pairwise model comparisons were performed using the DeLong test. Holm correction was applied to account for multiple comparisons. Learning curve analysis was conducted using stratified 5-fold cross-validation. Training AUC and validation AUC were calculated across increasing training sample sizes. Baseline continuous variables were summarized as either means or medians with their standard deviations or interquartile ranges, and categorical variables will be summarized as the number and percentage. Pairwise differences in AUCs among models were evaluated using DeLong's test, and the Holm method was applied to adjust for multiple comparisons. Baseline characteristics were compared between the fragility fracture and non-fragility fracture groups. Continuous variables were first assessed for normality using the Shapiro-Wilk test. Normally distributed continuous variables were summarized as mean ± standard deviation and compared using the independent-samples *t* test or Welch's *t* test, as appropriate. Non-normally distributed continuous variables were summarized as median and interquartile range and compared using the Mann-Whitney U test. Categorical variables were summarized as counts and percentages and compared using Pearson's chi-square test or Fisher's exact test, as appropriate. To assess the potential influence of multiple baseline comparisons, Holm-adjusted and Benjamini-Hochberg false discovery rate-adjusted *p* values were additionally calculated. Baseline comparisons were considered descriptive and exploratory, and clinically relevant or statistically imbalanced variables were considered in subsequent analyses.

## 3 Results

### 3.1 Characteristics of participants

In the comparison of baseline characteristics, significant differences were observed between the fragility fracture group (*n* = 961) and the non-fragility fracture group (*n* = 164) across multiple variables (Supplementary Table 1). Regarding gender distribution, the proportion of females was higher in the fragility fracture group (79.9%) compared with the non-fragility fracture group (68.9%), while the proportion of males was relatively higher in the latter (31.1% vs. 20.1%, *p* = 0.002). Because the continuous variables did not satisfy the normality assumption, they were summarized as median and interquartile range [median (IQR)] and compared using the Mann-Whitney U test. In terms of laboratory indicators, significant differences were found in hepatitis C virus IgG antibody values, fibrinogen, D-dimer, total bile acid, and serum sodium levels between the two groups (*p* < 0.001). The fragility fracture group showed higher D-dimer levels, higher fibrinogen levels, higher total bile acid levels, and lower serum sodium levels. Bone mineral density-related indicators demonstrated that both lumbar spine T-scores and hip T-scores were significantly lower in the fragility fracture group (*p* < 0.001), suggesting lower bone mineral density in this group. Regarding demographic characteristics, the fragility fracture group had a significantly higher median age [75.00 (69.00, 81.00) years vs. 66.50 (60.00, 73.00) years, *p* < 0.001], as well as lower body weight and BMI. Height did not differ significantly between the two groups. Overall, the two groups showed significant imbalances in gender, age, body weight, bone mineral density, and several blood biochemical parameters, which warrant consideration in subsequent analyses.

### 3.2 Evaluation of different feature selection methods

**Figure 2:**
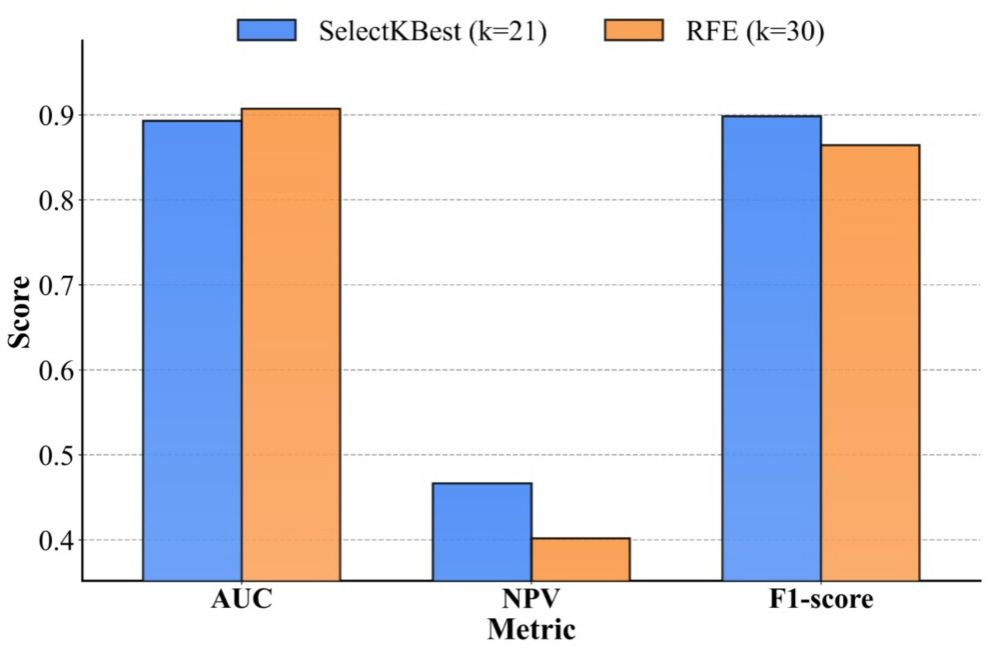
The performance of two selection methods on the training dataset is being evaluated.

Too many features might introduce noise and some irrelevant information into the dataset, which in some cases makes training more complex and makes it hard for the algorithm to spot the key predictors. To check whether RFE could actually handle these issues, we thought a comparative study against SelectKBest would be useful. In our test, RFE picked 30 features while SelectKBest picked 21. For the evaluation, we used ten-fold cross-validation, a method that provides reliable estimates because every subset of data is used for both training and validation, which might suggest the results are more solid. We compared the results from both methods, and the details are shown in Figure 2. In our study, it likely shows that RFE has some advantages and gives us a better idea of how different feature selection strategies can impact overall performance, although that could depend on the specific situation.

### 3.3 Comparison of different machine learning algorithms

**Figure 3:**
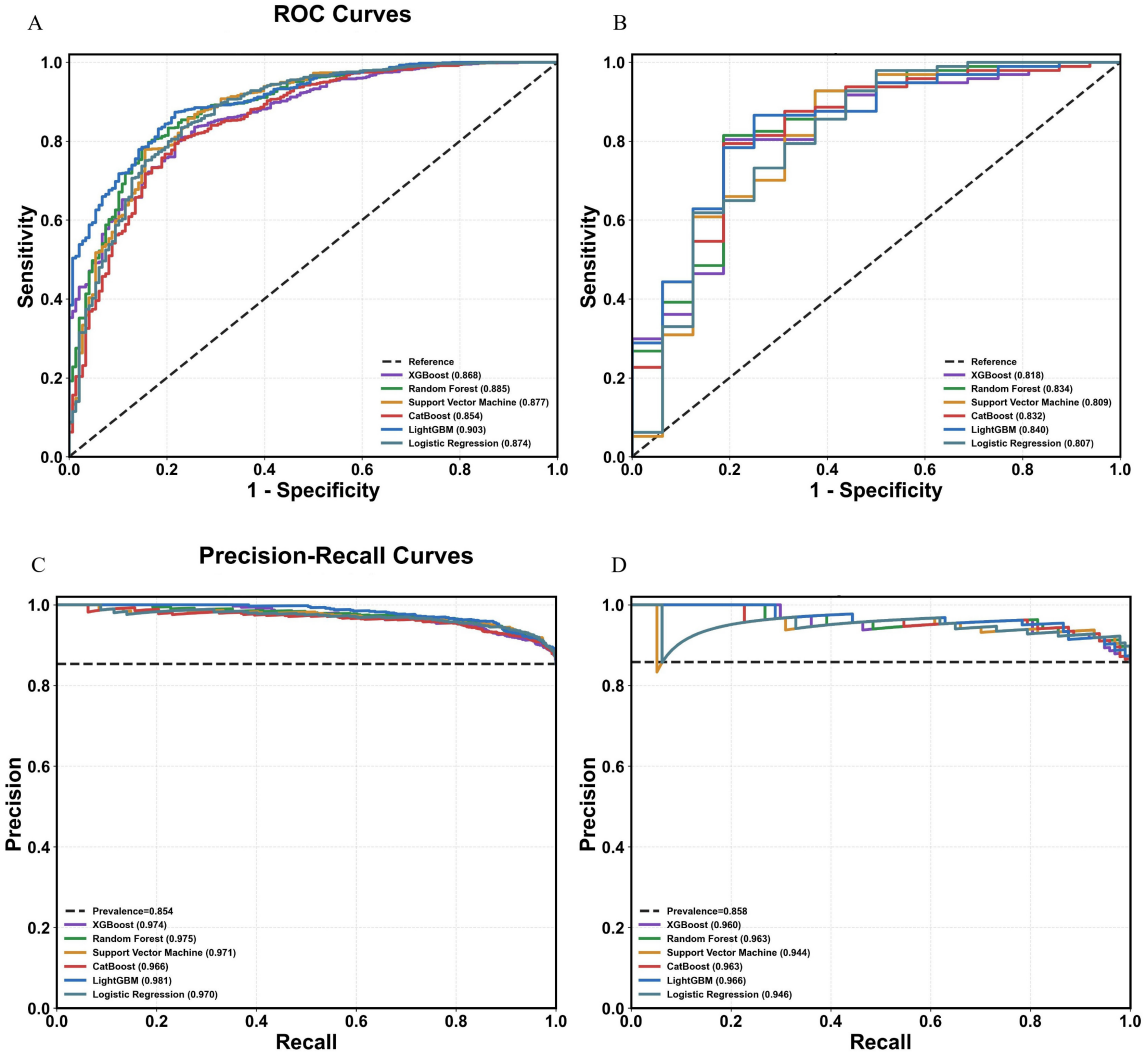
Discrimination performance of six machine-learning models. Receiver operating characteristic (ROC) curves for **(A)** train and **(B)** test cohorts, and precision–recall (PR) curves for **(C)** train and **(D)** test cohorts across XGBoost, Random Forest, SVM, CatBoost, LightGBM and Logistic Regression.

**Figure 4:**
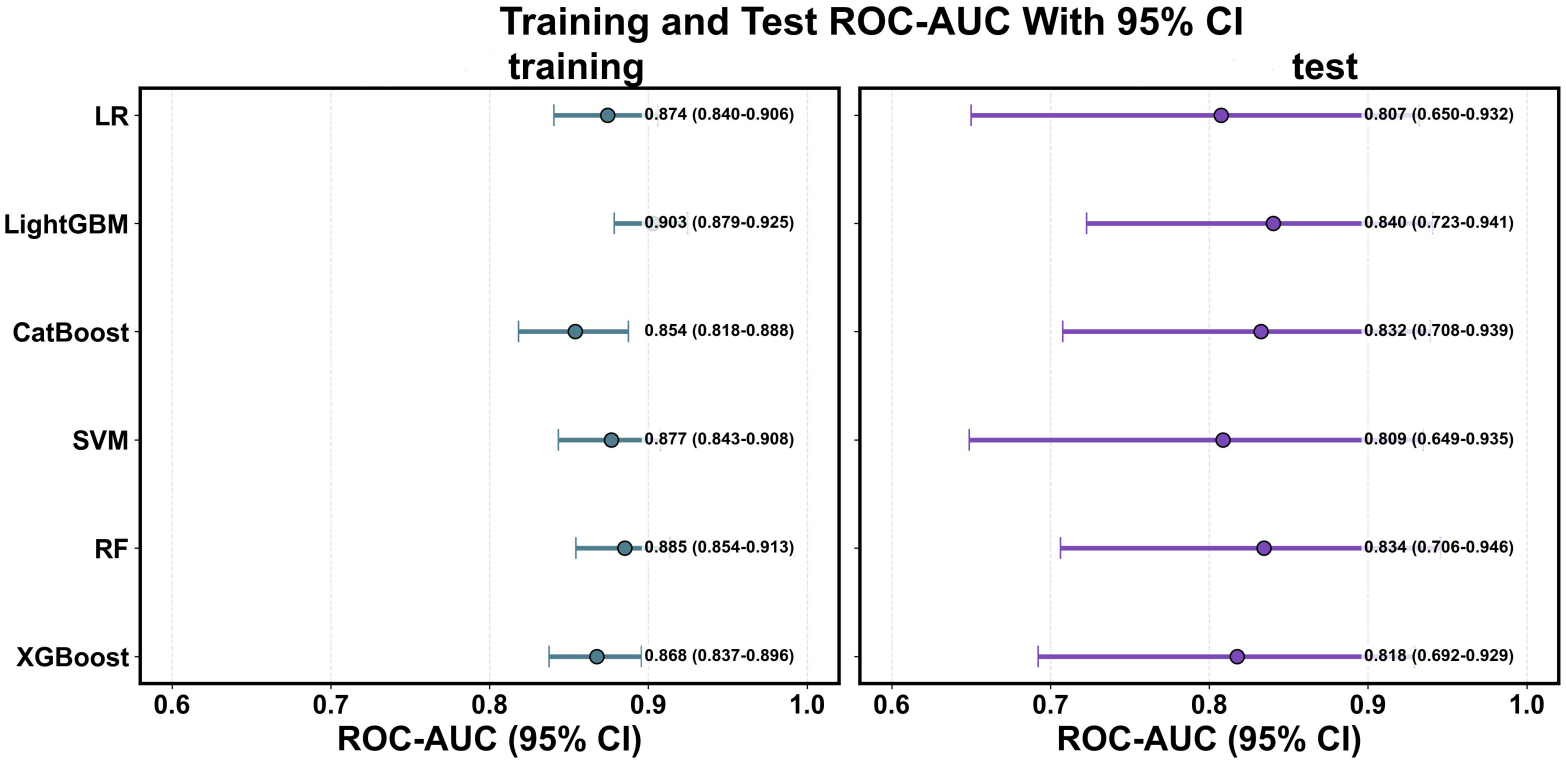
Train and test ROC-AUC with 95% confidence intervals. Forest plot summarizing train AUC and test AUC for each model with corresponding 95% CIs to visualize uncertainty and train–test performance shifts.

**Figure 5:**
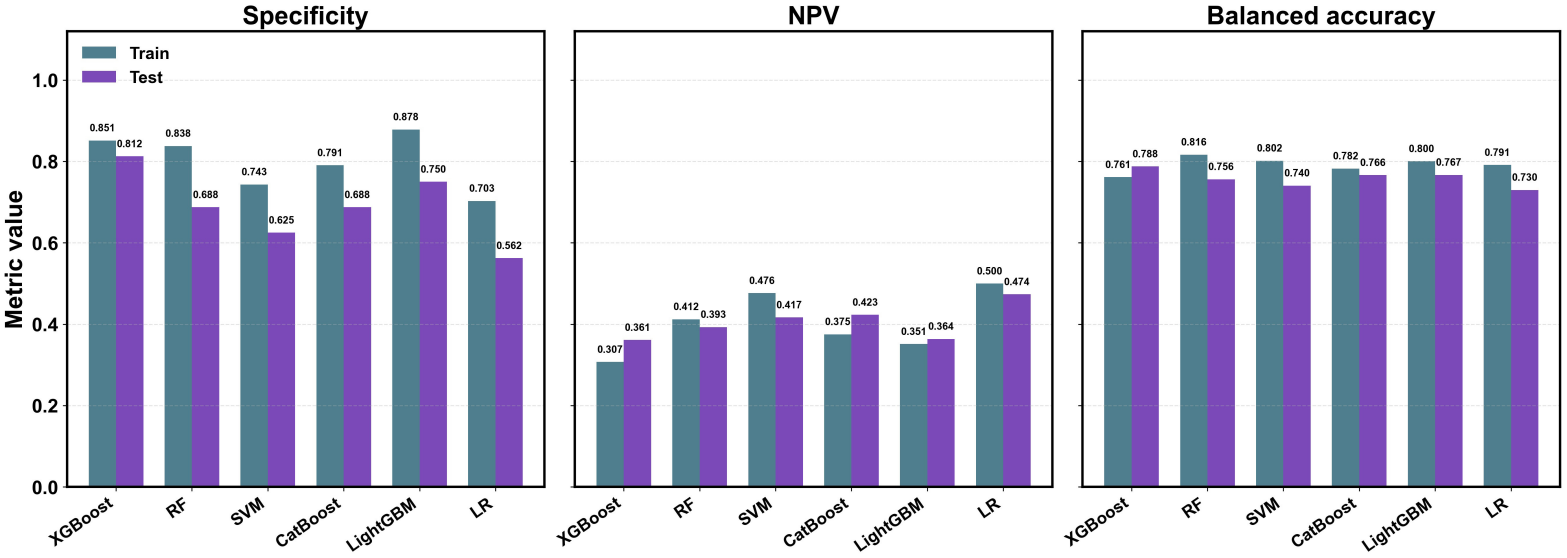
Bar plots comparing train vs. test performance for specificity, negative predictive value (NPV) and balanced accuracy across the six models using a fixed threshold derived from training out-of-fold predictions.

**Figure 6:**
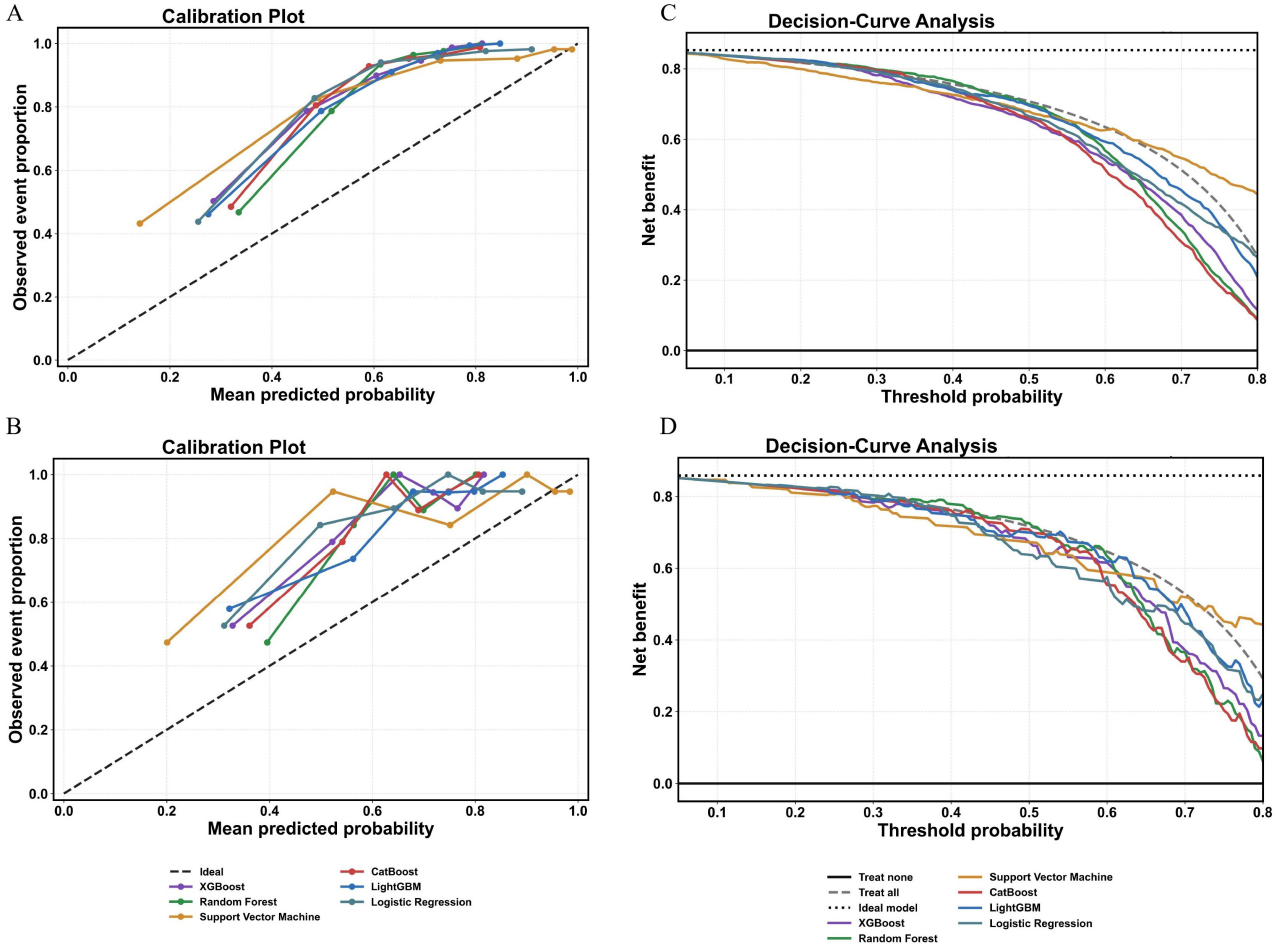
Calibration and decision-curve analysis of predicted fracture risk. Calibration plots for **(A)** train and **(B)** test cohorts, and decision-curve analysis (DCA) for **(C)** train, and **(D)** test cohorts across the six models.

We evaluated six machine learning algorithms, including XGBoost, Random Forest, SVM, CatBoost, LightGBM, and Logistic Regression. Given the clinical context of fragility-fracture prediction, model performance was not assessed by ROC-AUC alone (Figure 3). In addition to ROC-AUC and its 95% confidence interval (Figure 4), we reported PR-AUC (Figure 3), specificity, negative predictive value, balanced accuracy (Figure 5). Calibration plots and decision-curve (Figure 6) analysis were further used to examine probability calibration and potential clinical utility across different threshold probabilities.

In the independent test cohort, the six models showed broadly comparable discrimination. LightGBM achieved the highest ROC-AUC of 0.840 (95% CI, 0.727–0.936), followed by Random Forest. LightGBM also showed the highest PR-AUC (0.966), together with a relatively low Brier score (0.133), specificity of 0.750 and balanced accuracy of 0.767 (Supplementary Table 2). These findings suggest that LightGBM provided a favorable balance between discrimination, calibration and threshold-dependent performance. Random Forest and CatBoost performed similarly well, with test-set AUCs of 0.834 and 0.832 and PR-AUCs of 0.963 and 0.963, respectively. CatBoost showed the smallest train-test AUC gap, whereas Random Forest achieved slightly higher discrimination than CatBoost in the test cohort. XGBoost had a test-set AUC of 0.818 and PR-AUC of 0.960. Although its overall discrimination was slightly lower than that of LightGBM, it achieved the highest specificity (0.813) and balanced accuracy (0.788), indicating comparatively strong performance in distinguishing fracture from non-fracture cases at the selected threshold. Logistic Regression and SVM showed higher sensitivity, but their lower specificity suggested a greater tendency to classify patients as positive in this imbalanced clinical setting.

**Figure 7:**
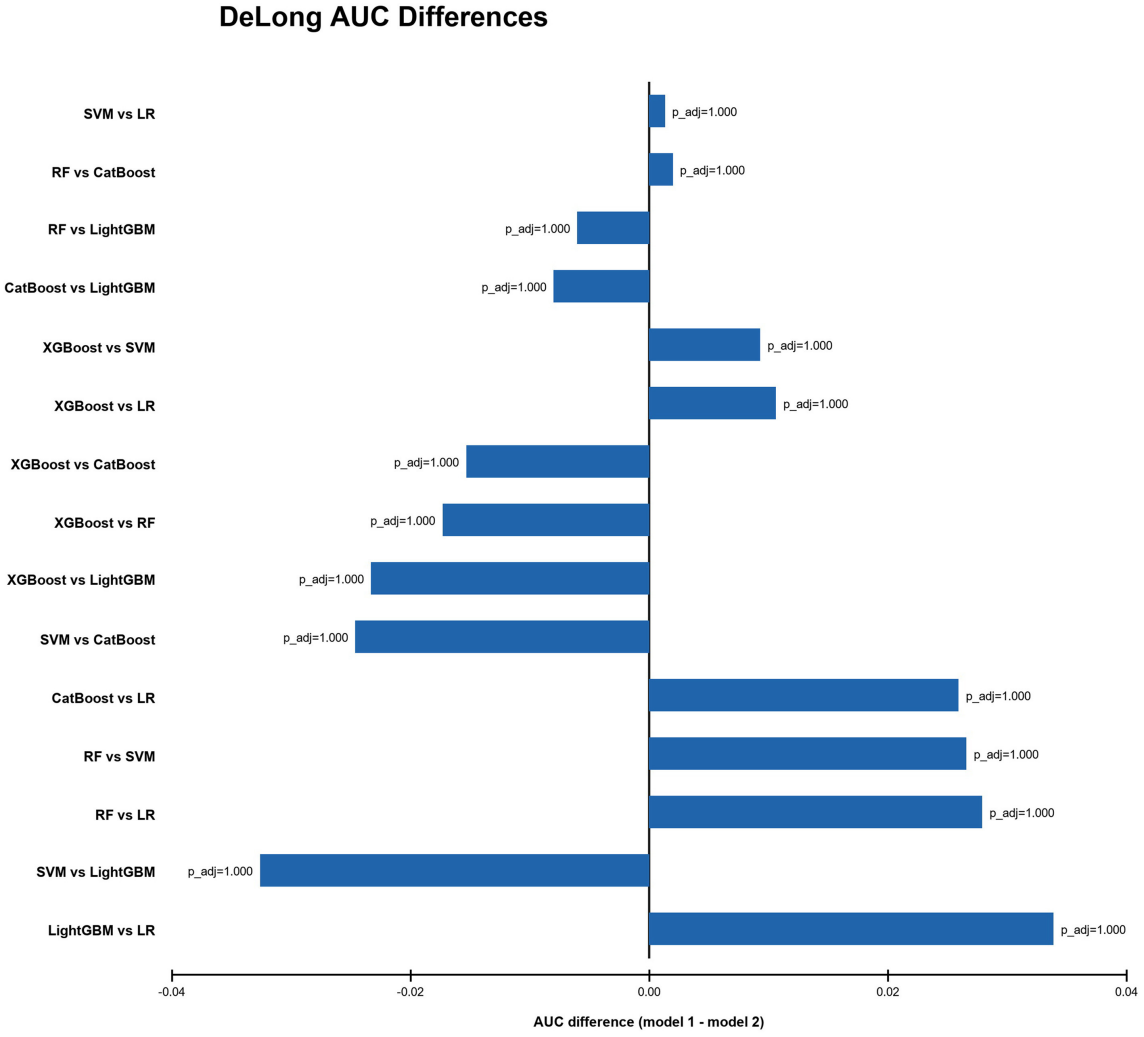
Pairwise differences in test AUC estimated by DeLong's test, with Holm correction for multiple comparisons; positive values indicate higher AUC for the first-listed model.

Pairwise DeLong tests with Holm correction were performed on test data to compare the ROC-AUCs of the six models (Figure 7). No statistically significant pairwise differences were observed after correction, indicating that the discrimination performance of the models was statistically comparable. Therefore, the preferred model was not chosen solely on the basis of nominal AUC ranking. Instead, model selection considered the overall pattern of ROC-AUC, PR-AUC, calibration, decision-curve analysis. On this basis, LightGBM was selected as the preferred model for subsequent interpretation, as it showed the most favorable overall profile across discrimination, calibration and robustness criteria.

### 3.4 Importance of the selected features

**Figure 8:**
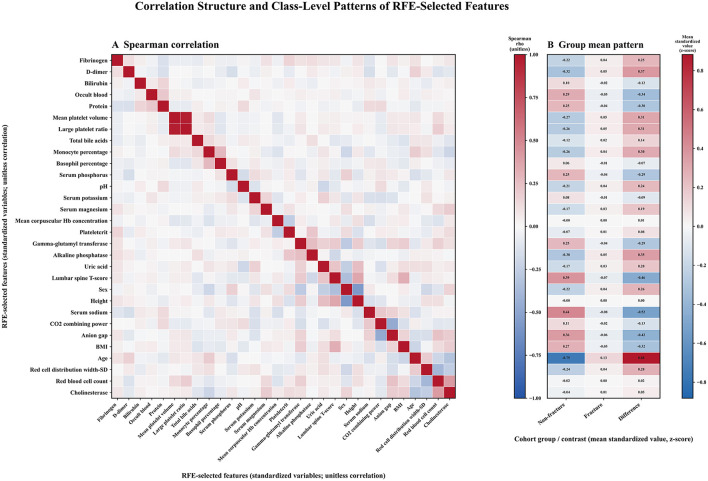
Correlation structure and class-level patterns of RFE-selected features. **(A)** Spearman correlation heatmap among the RFE-selected features and **(B)** group-mean pattern heatmap contrasting fracture vs. non-fracture cohorts, highlighting feature interdependencies and class-level shifts.

To investigate the interrelationships among the 30 key features selected via Recursive Feature Elimination (RFE), a Pearson correlation heatmap was constructed (Figure 8). Fibrinogen is a soluble glycoprotein that plays an essential role in both coagulation and inflammation, which is closely linked with inflammatory markers (27). These inflammatory signals accelerate bone loss by promoting resorption and inhibiting formation, thereby increasing fracture susceptibility (28). It has been reported that the bone mass status displayed a positive correlation with fibrinogen levels (29). This study shows an inconsistency with our result. D-dimer is a coagulation biomarker, which is the degradation product of fibrinogen, and had been reported to be elevated in patients with fractures (30). D-dimer had a significant effect at pre- and post-operation in elderly osteoporotic hip fracture patients (31). Besides, the BMD of Wards triangle was negatively related to D-dimer levels (29). Patients with HCV infection might be at an increased risk of osteoporosis (32).reduced BMD is common in this population of chronic HCV-infected patients (33). High sodium intake increases calcium excretion, which in turn is associated with lower bone mineral density (BMD), a marker of osteoporotic fracture risk (32). This has led to the hypothesis that sodium could be a dietary risk factor for osteoporosis (34). Total bile acids, a component of signaling molecules that plays a role in metabolism of glucose and lipid (35). Studies have also identified that circulating Total bile acids was positively correlated with BMD, indicating the potential role of bile acids in the regulation of bone metabolism (36, 37). There are several potential causes for lumbar spine T-score, such as fracture risk (38).

The heatmap was used to characterize the RFE-selected feature panel in terms of inter-feature correlation and group-level distribution (Figure 8). Panel A shows the pairwise Spearman correlations among the 30 selected variables. Most correlations were weak to moderate, indicating limited redundancy within the selected feature set. Relatively stronger correlations were observed among several laboratory-related variables, including platelet-associated indices, suggesting that some features captured related clinical information. Panel B summarizes the standardized mean values of the selected features in the fracture and non-fracture groups. Compared with the non-fracture group, the fracture group showed higher standardized values for age, D-dimer, alkaline phosphatase, fibrinogen, mean platelet volume, large platelet ratio, and monocyte percentage. In contrast, lower standardized values were observed for serum sodium, lumbar spine T-score, BMI, anion gap, occult blood, protein, serum phosphorus, and gamma-glutamyl transferase.

Together, these results indicate that the RFE-selected features captured both partially independent information and measurable group-level differences between patients with and without fracture. These findings support the use of the selected feature panel for downstream predictive modeling, while remaining descriptive rather than causal in interpretation.

### 3.5 Learning curve analysis

**Figure 9:**
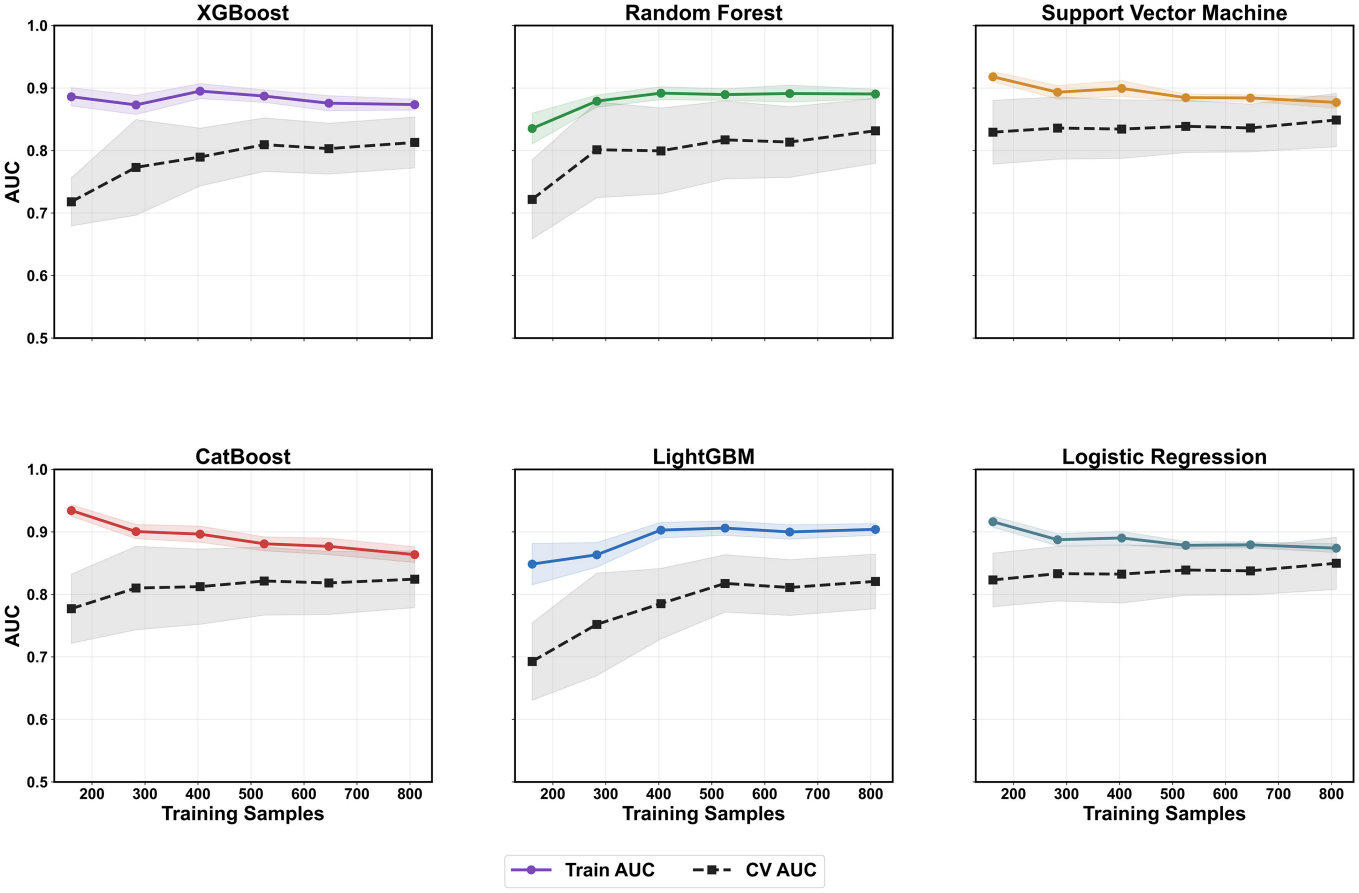
Learning-curve analysis for overfitting assessment. Learning curves showing train AUC and cross-validated (CV) AUC as a function of training sample size for the six models; shaded areas indicate variability across folds, supporting evaluation of generalization stability.

Learning-curve analysis further supported the stability of the revised modeling framework. As the training sample size increased, the cross-validated AUCs of most models gradually stabilized rather than showing progressive divergence between training and validation performance (Figure 9). The final train-validation AUC gaps were generally small, indicating that the models did not show evidence of severe overfitting. Although LightGBM showed a slightly larger final train-validation gap than some other algorithms, its independent test-set AUC remained the highest among the six models, and the apparent train-test AUC gap remained below the prespecified 0.07 threshold. These findings suggest that the selected modeling strategy achieved a reasonable balance between discrimination and generalizability.

### 3.6 Interpretation of LightGBM model via the SHAP method

**Figure 10:**
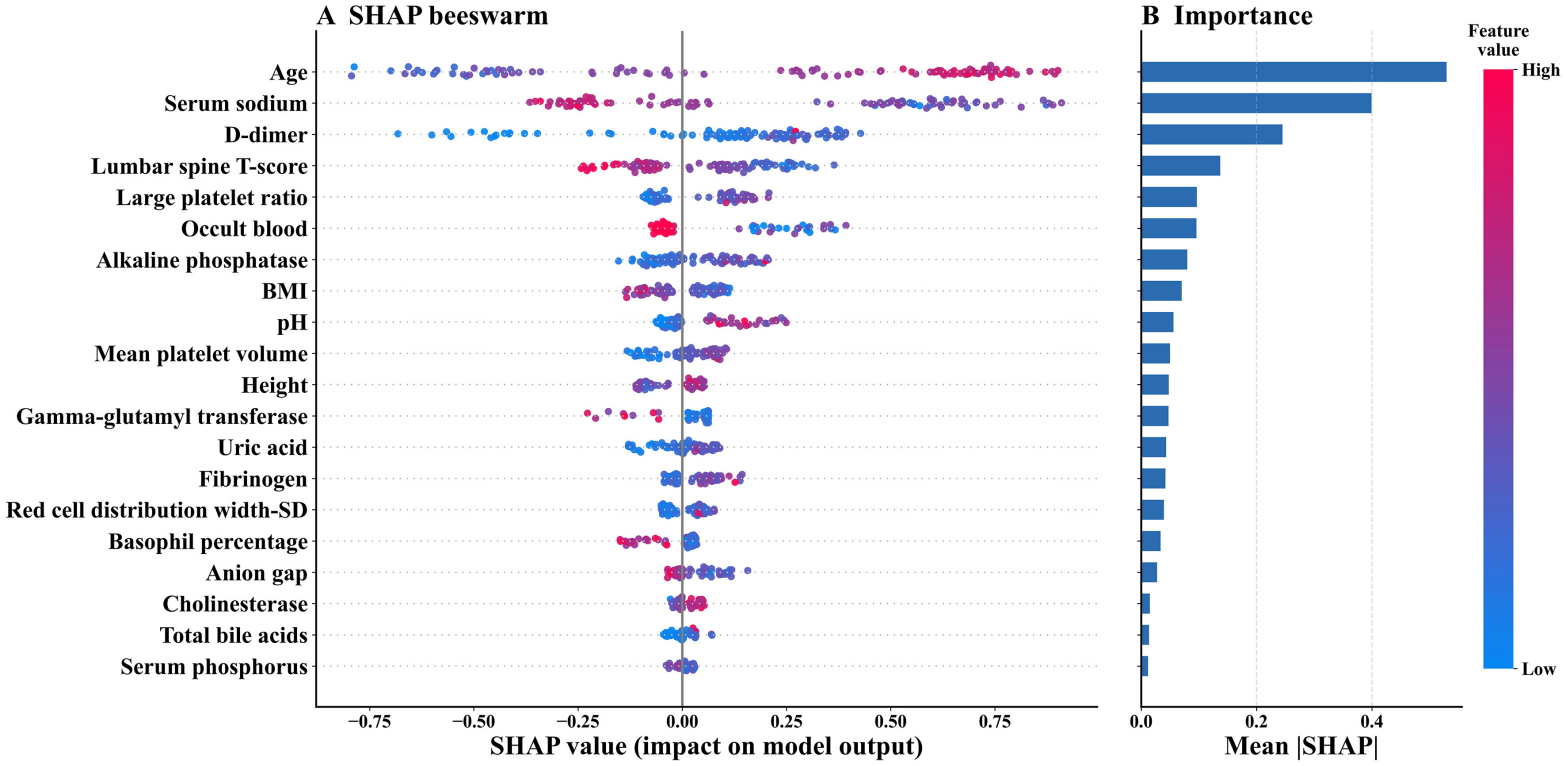
Model interpretation using SHAP for LightGBM. **(A)** SHAP beeswarm plot showing the distribution of feature contributions to model output and **(B)** mean absolute SHAP importance ranking for the top features.

The SHAP framework was utilized to interpret and quantify feature contributions to LightGBM predictions. Because the present study was retrospective and observational, SHAP values were interpreted as measures of contribution to model output rather than evidence of causal effects on fracture development. The analysis identified age, hepatitis C virus IgG antibody, and serum sodium as the most significant contributors to predicted fracture risk (Figure 10). Higher SHAP values for these features indicated that, within the fitted model, they shifted predictions toward a higher probability of fracture; however, this should not be interpreted as demonstrating that these variables directly caused fracture occurrence.

Conversely, lumbar spine and hip T-scores, which reflect bone mineral density showed negative SHAP contributions in many patients, indicating that higher T-scores tended to shift model predictions toward a lower probability of fracture. Additionally, elevated levels of biomarkers such as D-dimer, total bile acids, and fibrinogen were associated with increased model-predicted fracture probability. These variables may capture clinical status, comorbidity burden, inflammatory or metabolic perturbations, or treatment-related physiological changes, and they may also be correlated with other predictors included in the model. Therefore, their importance in the SHAP analysis should be interpreted as predictive relevance rather than confirmed biological or causal involvement in fragility fracture pathogenesis. Overall, the SHAP analysis provided a transparent description of how the model used available clinical and laboratory information to generate individualized fracture-risk predictions.

## 4 Discussion

This research undertakes a comprehensive examination of ML models to explore various factors linked to osteoporotic fragility fractures. ML, a widely-used computational approach, utilizes clinical data, imaging data and laboratory data. Rather than relying on discrimination alone, we evaluated model performance across complementary domains, including ROC-AUC with 95% confidence intervals, PR-AUC, specificity, negative predictive value (NPV), balanced accuracy, probability calibration and decision-curve analysis (DCA), to better reflect the asymmetric clinical consequences of false-positive and false-negative predictions.

Across the six candidate algorithms (XGBoost, Random Forest, SVM, CatBoost, LightGBM and Logistic Regression), discrimination performance was broadly comparable when accounting for uncertainty. Pairwise DeLong tests with Holm correction did not identify statistically significant differences in ROC-AUC between models, indicating that the observed ranking by point estimates should not be over-interpreted as definitive superiority. Accordingly, model selection was guided by the overall balance of test-set discrimination, PR-AUC, threshold-dependent clinical metrics (specificity/NPV/balanced accuracy), calibration behavior and robustness against overfitting, rather than ROC-AUC alone.

To mitigate potential optimistic bias, all preprocessing operations that can induce information leakage were confined to the training data. Specifically, oversampling (SMOTE), feature selection and hyperparameter tuning were performed within cross-validation folds, and the independent test cohort was held out from feature selection, tuning and threshold selection. Feature selection was examined using both SelectKBest and RFE under the same leakage-controlled pipeline. In our data, RFE tended to yield a more favorable generalization profile for the tree-based models, particularly LightGBM, whereas differences were smaller or inconsistent for other algorithms. Importantly, the final feature panel was fixed prior to test-set evaluation and applied consistently across models to ensure fair comparison.

From a clinical perspective, the augmented evaluation highlights why AUC alone is insufficient in imbalanced clinical-risk prediction. Models with similar ROC-AUC can still differ meaningfully in specificity, NPV and balanced accuracy at clinically relevant thresholds, and in the reliability of predicted probabilities. Calibration is particularly salient for fracture-risk stratification, because poorly calibrated probabilities can mislead downstream decisions even when discrimination appears acceptable. The DCA results provide an additional lens on potential clinical utility by quantifying net benefit across a range of threshold probabilities, complementing discrimination and calibration.

Interpretability analyses were used to characterize which variables most strongly influenced model predictions, not to assert causal risk factors. SHAP attributes model output to features under the fitted model and dataset distribution; therefore, SHAP-ranked variables should be interpreted as predictive contributors and proxies for underlying physiological or care-process states, rather than mechanistic drivers of fracture development. This distinction is essential in a retrospective inpatient setting, where laboratory markers may be correlated with each other and may also reflect acute illness, hospitalization, or peri-event physiological changes. The observed correlation structure among the selected variables supports this caution, with several laboratory features demonstrating non-trivial interdependencies.

Nevertheless, there are a couple of limitations to our study. To begin with, this is a retrospective study that is limited to one center (Yuebei People's Hospital) and so, selection bias may be a problem. External validity therefore remains uncertain, and prospective multicentre evaluation is required before considering clinical deployment. Second, although class imbalance was explicitly addressed using SMOTE within cross-validation, residual bias may persist, and performance may shift under different prevalences or care settings. Third, the modest size of the independent test cohort implies that uncertainty intervals can be wide; we therefore report 95% CIs and avoid over-claiming small performance differences.

In order to verify the robustness of our model, future work will need multicenter, prospective data. Future studies should also examine temporal validation, recalibration strategies in new cohorts, and the impact of incorporating additional predictors (e.g., medication history, lifestyle factors, and longitudinal laboratory trajectories) that were not consistently available in the present dataset. Finally, mechanistic interpretation of SHAP-ranked variables should be pursued through targeted epidemiological or experimental designs rather than inferred from associative model explanations alone.

## 5 Conclusions

In conclusion, we developed an explainable machine learning approach for osteoporotic fragility fractures using six ML algorithms. Under a leakage-controlled evaluation pipeline with SMOTE, cross-validated threshold selection and test cohort, the candidate models achieved broadly comparable discrimination, and the preferred model was chosen based on the overall balance of discrimination, PR-AUC, clinically relevant threshold-dependent metrics, calibration and overfitting control rather than ROC-AUC alone. SHAP analysis helped us build a fracture risk model, which made it easier to identify high-risk populations.

## Data Availability

The raw data supporting the conclusions of this article will be made available by the authors, without undue reservation.

## Supplementary material

Supplementary Table 1 Baseline clinical, laboratory, and characteristics of patients with fragility versus non-fragility fractures.

Supplementary Table 2 Training out-of-fold and test-set performance of six machine-learning models for fragility fracture classification.
